# Conformational selection and induced fit for RNA polymerase and RNA/DNA hybrid backtracked recognition

**DOI:** 10.3389/fmolb.2015.00061

**Published:** 2015-11-05

**Authors:** Jian Wu, Wei Ye, Jingxu Yang, Hai-Feng Chen

**Affiliations:** ^1^State Key Laboratory of Microbial Metabolism, Department of Bioinformatics and Biostatistics, College of Life Sciences and Biotechnology, Shanghai Jiaotong UniversityShanghai, China; ^2^Shanghai Center for Bioinformation TechnologyShanghai, China

**Keywords:** backtracked RNA polymerase, DNA/RNA hybrid, induced fit, conformational selection, *P*-test

## Abstract

RNA polymerase catalyzes transcription with a high fidelity. If DNA/RNA mismatch or DNA damage occurs downstream, a backtracked RNA polymerase can proofread this situation. However, the backtracked mechanism is still poorly understood. Here we have performed multiple explicit-solvent molecular dynamics (MD) simulations on bound and apo DNA/RNA hybrid to study backtracked recognition. MD simulations at room temperature suggest that specific electrostatic interactions play key roles in the backtracked recognition between the polymerase and DNA/RNA hybrid. Kinetics analysis at high temperature shows that bound and apo DNA/RNA hybrid unfold via a two-state process. Both kinetics and free energy landscape analyses indicate that bound DNA/RNA hybrid folds in the order of DNA/RNA contracting, the tertiary folding and polymerase binding. The predicted Φ-values suggest that C7, G9, dC12, dC15, and dT16 are key bases for the backtracked recognition of DNA/RNA hybrid. The average RMSD values between the bound structures and the corresponding apo ones and Kolmogorov-Smirnov (KS) *P*-test analyses indicate that the recognition between DNA/RNA hybrid and polymerase might follow an induced fit mechanism for DNA/RNA hybrid and conformation selection for polymerase. Furthermore, this method could be used to relative studies of specific recognition between nucleic acid and protein.

## Introduction

RNA polymerase efficiently catalyzes the template-directed transcription with a low error rate (Shaevitz et al., 2003). This high fidelity may be the result of a “proofreading” mechanism. Backtracked transcribing complex plays a key role in the proofreading mechanism of transcription (Jeon and Agarwal, 1996; Thomas et al., 1998; Shaevitz et al., 2003; Kostrewa et al., 2009; Wang et al., 2009; Martinez-Rucobo et al., 2011). RNA polymerase does not move forward monotonously. It oscillates between three stable states to move forward or backward at every step in transcription (Thomas et al., 1998; Toulmé et al., 1999; Darzacq et al., 2007; Wang et al., 2009). If the forward movement is blocked, for example, by DNA/RNA mismatch or DNA damage, a backtracked state of the transcribing complex will form. Then the transcription blocking may be recovered by transcription factor SII (TFIIS) in eukaryotes and GreA (and/or GreB) in prokaryotes (Wind and Reines, 2000; Fish and Kane, 2002; Shilatifard et al., 2003; Sims et al., 2004).

Besides naturally occurrence, ultraviolent light, toxic chemical agents, and ionizing radiation could cause DNA damage with a relatively higher possibility (Jackson and Bartek, 2009). DNA damage would lead to many kinds of cancers (Jackson and Bartek, 2009). In addition, DNA damage and its repair are important hypotheses of aging mechanism (Hoeijmakers, 2009). There are several ways of DNA damage repair, including nucleotides excision induced by backtracked RNA polymerase II (Hanawalt, 2002; Fousteri and Mullenders, 2008; Wang et al., 2009), cell cycling arresting or apoptosis induced by p53 (Wang, 1998; Cortez et al., 2001; Kruse and Gu, 2009) or Chk2 (Lim et al., 2000; Li and Zou, 2005). Therefore, it is critical to reveal the specific recognition between the backtracked RNA polymerase II and damaged DNA/RNA hybrid.

In the backtracked state, RNA polymerase retreats on the template, extruding the 3′-end of the RNA (Wang et al., 2009). In eukaryotes, TFIIS binds to backtracked RNA polymerase II and stimulates a ribonuclease activity which cuts off the 3′-end of nascent RNA. As a result, RNA polymerase II elongation complex can repeatedly shorten and extend the nascent RNA, which is referred as cleavage and resynthesis (Reines et al., 1993). Therefore, RNA polymerase timely recovers the blocked transcription.

**Figure 1:**
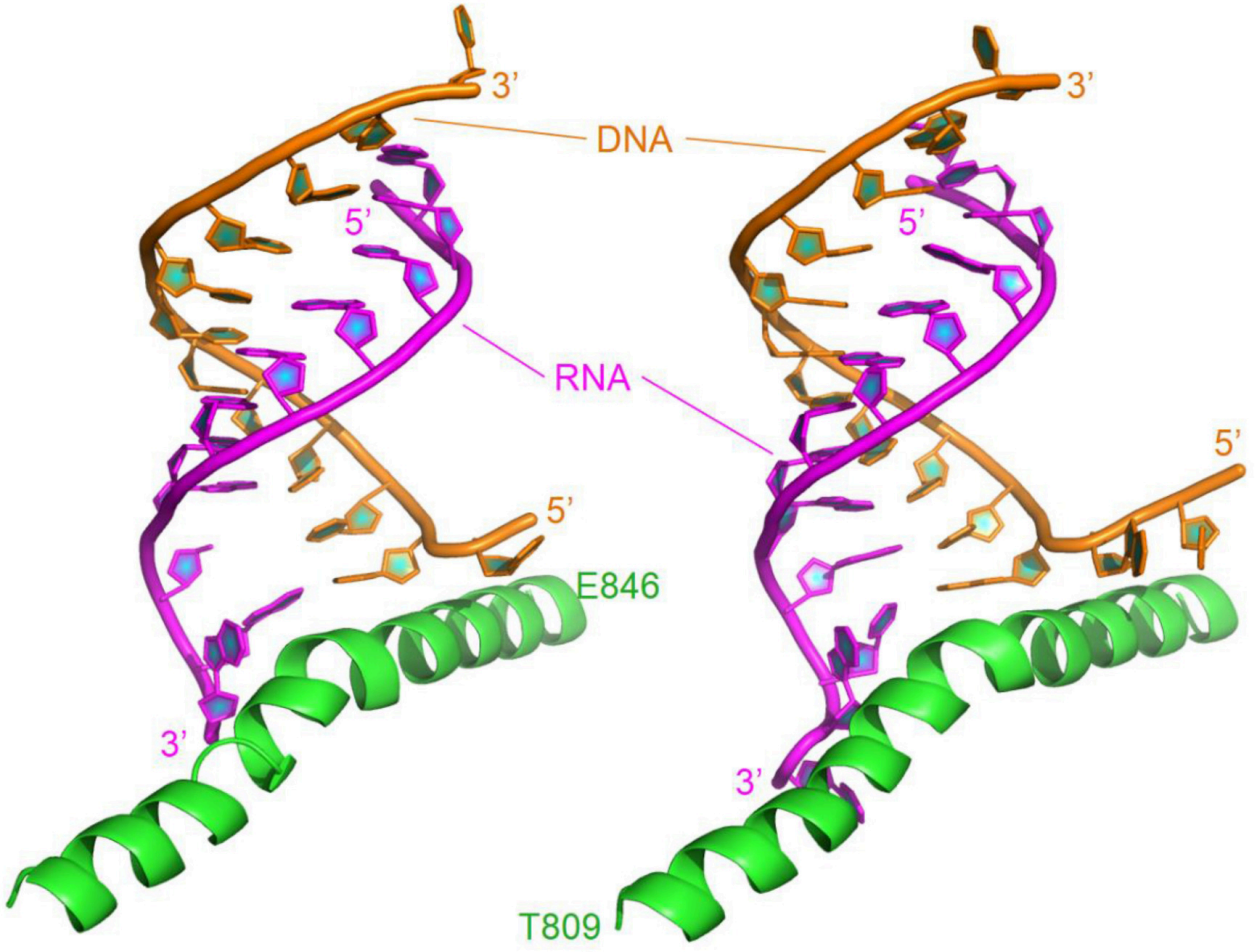
**Ribbon representation of the original structures of bound DNA/RNA hybrids with DNA damage (left) and DNA/RNA mismatches (right)**. Polymerase is in green, DNA in orange, and RNA in magenta.

The X-ray structure of backtracked RNA polymerase II and DNA-RNA hybrid complex was released in 2009 (pdb code: 3GTQ) (Wang et al., 2009). The complex in this study consists of one chain of damaged DNA, one chain of 11 nt RNA, and one bridge helix from Thr809 to Glu846. The bridge helix is bound to the 3′-end of the RNA. In order to compare the difference between DNA damage and DNA mismatch, the complex between RNA polymerase II and mismatched DNA-RNA hybrid (pdb code: 3GTK) was also investigated. The complex includes 13 nt of the RNA with two mismatches at its 3′-end (C-dC and G-dG), 13 nt of the DNA, and the bridge helix (shown in Table 1 and Figure 1).

**Table 1:** **Information of DNA-RNA hybrid and polypeptide**.

**Model**		**DNA-RNA hybrid**	**Polypeptide**
DNA/RNA hybrid with DNA damage	Apo	1----6----11 RNA 5′ AUGGCGCGGAG 3′ ||||||||||| DNA 3′ CTACCTCTCCTC 5′ --21---16---	/
	Bound		809--814--819--824-H_2_N^+^-TPQEFFFHAMGGREGLIDTAVKTAETGYIQRRLVKALE-COO^−^-829--834--839--844
	Protein	/	
Bound DNA/RNA hybrid with mismatches		1----6----11- RNA 5′ AUCGAGAGGAUGC 3′ |||||||||||:: DNA 3′ TAGCTCTCCTAGC 5′ 26—21—16-	

X-ray experiments suggest that the uridine 5′-monophosphate base of RNA was paired with its complement in the DNA template but tilted 15°–20° out of the plane. The backbone of the backtracked RNA between the last match base and the mismatched base was bent over 120° out of the path of the hybrid helix (Wang et al., 2009). Therefore, mismatched RNA/DNA undergoes significant conformational adjustment through specific recognition. However, the recognition between RNA/DNA hybrid and polymerase is poorly understood. Molecular dynamics (MD) simulation may be a powerful tool for analyzing the structural and dynamic features of biomacromolecules. Several simulations were performed on RNA polymerase II. Suenaga et al. (2006) did whole molecule MD simulation on normal RNA polymerase II and DNA/RNA hybrid to discover the elongation mechanism in 2006. The transcription bubble is formed and maintained by multiple protein loops. MD simulation on backtracked RNA polymerase II and mismatched DNA/RNA hybrid was mentioned in work of Wang et al. (2009). The two nanoseconds' simulation reveals that the mismatched bases are highly mobile and unstable. Here we focus on the folding kinetics of DNA/RNA hybrid upon polymerase-binding. Our research intends to reveal a series of interesting questions. (1) How does the conformation of the DNA/RNA hybrid change upon the binding of RNA polymerase II? (2) Which mechanism does the folding of DNA/RNA and polymerase obey? (3) How does the enzyme recognize the DNA damage site of the DNA/RNA hybrid? To answer these questions, we utilize atomic molecular dynamics simulation in explicit solvent to analyze the coupling mechanism between DNA/RNA folding and polymerase binding.

However, all atomic MD simulations are currently restricted to the timescale of less than 1 μs, which is much shorter than the folding half-time of most biomacromolecules at room temperature (at least 1 ms) (Baker, 1998; Fersht and Daggett, 2002). Fortunately, the rate of unfolding can accelerate by approximately six orders of magnitude at high temperature (usually 498 K) (Shea and Brooks, 2001), so most biomacromolecules unfold in the nanosecond's time scale at this temperature (Fersht and Daggett, 2002). Furthermore, experiment confirms the transition state for folding and unfolding is expected to be the same from the principle of microscopic reversibility (Fersht and Daggett, 2002). Based on these previous works, unfolding simulations at high temperature have been used in the current study. In this paper, we carried out multiple molecular dynamics ran in explicit water (Interlandi et al., 2006) to study the folded states and the unfolding kinetics for both bound and apo-states to understand the specific recognition between DNA/RNA hybrid and RNA polymerase.

## Methods

### Molecular dynamics simulations

The atom coordinates of DNA/RNA-polymerase complex were obtained from the Protein Data Bank, with the accessory code 3GTQ (Wang et al., 2009). All simulations and most analyses procedures were conducted using the AMBER11 software package (Case TAD TEC et al., 2010). Hydrogen atoms were added using the LEaP module of AMBER11. Counter-ions were used to maintain system neutrality. All systems were solvated in a truncated octahedron box of TIP3P waters with a buffer of 10 Å. Particle Mesh Ewald (PME) (Darden et al., 1993) was employed to treat long-range electrostatic interactions with the default setting in AMBER11. The improved parm99SBildn force field (Lindorff-Larsen et al., 2010) was used for the intramolecular interactions. The SHAKE algorithm (Ryckaert et al., 1977) was used to constrain bonds involving hydrogen atoms. 1000-step steepest descent minimization was performed to relieve any structural clash in the solvated systems. This was followed by heating up and brief equilibration for 20 ps in the NVT ensemble at 298 K with PMEMD of AMBER11. Langevin dynamics with a time step of 2 fs were used in the heating and equilibration runs with a friction constant of 1 ps^−1^.

Folded state can be used to compare conformational differences between different systems, to determine inter-/intrachain interactions and to predict the recognition mechanism. A previous work suggests that a small number of trajectories of MD simulation (5–10) is sufficient to capture the average properties of the protein (Day and Daggett, 2005). Therefore, to study the folded state of each solvated system, six independent trajectories of 10.0 ns each in the NPT ensemble at 298 K were then simulated with PMEMD of AMBER8. Six independent trajectories of 15.0 ns each were performed for the apo-DNA/RNA, apo-polymerase, and DNA/RNA-polymerase complex in the NVT ensemble at 498 K to investigate the unfolding kinetics of each solvated system. Trajectories of 450 ns total were collected for three systems (DNA/RNA-polymerase complex, apo-DNA/RNA, and apo-polymerase) at both 298 K and 498 K, taking about 41,000 CPU hours on the Xeon (3.0 GHz) cluster. Detailed simulation information was listed in Table 2.

**Table 2:** **Simulation condition for five systems**.

**Model**		**Number traj**	**Simulation time (ns)**		**Water molecules number**	**Water box size (Å^3^)**	**Initial density (g/mL)**
			**298 K**	**498 K**			
**DNA damage**	**Bound**	**6**	**10.0**	**15.0**	**12789**	**445921**	**0.903**
	**Apo**	**6**	**10.0**	**15.0**	**6572**	**237294**	**0.883**
	**Protein**	**6**	**10.0**	**15.0**	**8566**	**158607**	**0.894**
**Mismatch**	**Bound**	**6**	**10.0**	**–**	**11779**	**418192**	**0.895**
	**Apo**	**6**	**10.0**	**–**	**7933**	**283023**	**0.890**

Native contacts between DNA/RNA hybrid and polymerase were monitored to determine the unfolding kinetics. Kinetics analysis suggested that 10 ns at 498 K were needed to reach the equilibrium stage for the bound and apo states, so that the first 10 ns (a total of 60 ns) were used to study the unfolding kinetics.

### Data analysis

Tertiary contact assignment was handled with in-house software (Chen and Luo, 2007; Chen, 2008; Qin et al., 2012; Ye et al., 2012a,b). Residues and nucleotides are in hydrophobic contact when mass centers of their side chains are closer than 6.5 Å for the complex. Electrostatic (i.e., charge-charge) interactions are assigned when the distance between the mass center of positive charge residue and the DNA/RNA hybrid phosphate backbone is less than 11 Å. Hydrogen bond is defined that the distance between two polar heavy atoms either of which has a hydrogen atom are less than 3.5 Å. The non-adjacent nucleotides and residues are in contact (termed “native contact”) when their side chains were closer than 7.5 Å. All the 3D molecular representations were visualized and rendered with PyMOL 1.5.

The energy landscape was performed by calculating normalized probability from a histogram analysis (Pande and Rokhsar, 1999). Here we used RMSD and the radius of gyration (Rg) to map the energy landscape. Unfolding kinetics were reflected and fitted in 2 indices: (1) Qf, the fraction of intra-chain (tertiary) native contacts in unfolding state, comparing to folded state (unfolding kinetics); and (2) Qb, the fraction of inter-chain native contacts (unbinding kinetics). Binding and folding pathways could be restored as a reverse procedure of unfolding pathway. The free energy landscapes and unfolding kinetics was plotted and fitted in Origin 8.6.

Landscapes were also used to analyze the distance differences to detect the structural changes between bound and apo-DNA/RNA hybrid. Distance between every pair of C5′ (DNA/RNA) or Cα (protein) was calculated in both bound and apo structure, respectively. Then the landscape of base-to-base (or residue-to-residue) distances in apo-structure minus the corresponding distances in bound structure was plotted. This indicates a structural contraction when the color in red is positive and an extension when the color in blue is negative. In order to exclude the effect of thermal fluctuations, we averaged all the distances in the whole 10 ns' simulation instead of a certain time point.

According to the definition of protein Φ-values which computed with a strategy similar to those used in other studies (Caflisch and Karplus, 1994; Vendruscolo et al., 2001; Gsponer and Caflisch, 2002), this method might be suitable for the Φ-values calculation for DNA/RNA hybrid. Therefore, the equation below was used to process the Φ-values of DNA/RNA hybrid.

$$\begin{array}{l} \Phi_{\text{DNA}∕\text{RNA}}^{calc}=\frac{N_{i}^{\text{TS}}-N_{i}^{\text{U}}}{N_{i}^{\text{F}}-N_{i}^{\text{U}}} \end{array}$$

$N_{i}^{TS}$ is the number of native contacts for base *i* at transition state, $N_{i}^{F}$ and $N_{i}^{U}$ is the number of native contacts for base *i* at folded and unfolded states, respectively.

### Binding mechanism evaluation

There are two mainstream hypotheses to explain binding process: “Induced fit” model (Koshland, 1958; Boehr and Wright, 2008) and “Conformational selection” model (Ma et al., 1999, 2002; Kumar et al., 2000; Tsai et al., 2001; Boehr and Wright, 2008; Tsai and Nussinov, 2011). The former model emphasizes that the conformational change of one part in the complex is induced by the binding of the other part (binding first, Turjanski et al., 2008), while the latter points out that one part selects the most optimal ligand among several chaperones with different conformations (Lange et al., 2008). If the folding obeys a mechanism of induced fit, a significant conformational change must occur upon the ligand binding, especially near the binding site. Otherwise, if the conformational change is not so significant, the binding tends to obey a conformational selection mechanism (Wlodarski and Zagrovic, 2009). Recent studies have demonstrated that the binding process may employ a mixed mechanism, selecting an optimal conformation globally and inducing the local structure of the ligand to be adjusted (Anthis et al., 2011; Bucher et al., 2011; Silva et al., 2011). Following the concepts above, induced fit could be calculated from the RMSD between a bound structure and its most “like” apo-structure (which has the lowest RMSD from the bound structure). In the induced-fit model, relative magnitude of conformational selection could be characterized from the average RMSD between this most similar apo-structure and the other apo-structures (Wlodarski and Zagrovic, 2009). In this study, binding site for the ligand was defined as the mass center of the receptor. RMSD for induced fit of 6 trajectories was given as a function of distance from the binding site. Because the RMSD values are not normally distributed, which is not suitable for a *t*-test, a standard two-sample Kolmogorov-Smirnov (KS) test was used to evaluate the statistical significance of RMSDs' variations. The equation below was used to quantitatively describe relative differences between conformational selection and induced fit.

$$△=\frac{\sum_{X_{i},f_{i}\in CS}X_{i}f}{N_{\text{CS}}}-\frac{\sum_{X_{j},f_{j}\in IF}X_{j}f_{j}}{N_{\text{IF}}}$$

*X* is the RMSD value and *f* is the frequency for a particular RMSD value region in a distribution of conformational selection (CS) or induced fit (IF), and *N*_*CS*_ and *N*_*IF*_ are the numbers of data points for the distribution of CS or IF, respectively.

### Transition state identification

The transition state reflects a representative snapshot at the free energy maxima of the unfolding and folding pathways. The structures at the free energy maxima constitute the transition state ensemble (TSE). TSE structures can either fold or unfold, and the transition probability (*P*) will be 50%. In order to determine TSE, we have scanned MD snapshots for TSE structures in all six unfolding trajectories for each of bound DNA/RNA and apo-DNA/RNA, respectively (Pande and Rokhsar, 1999). The transition probability curves are further fitted by the Boltzmann Equation,

$$\begin{array}{l} P=\frac{1}{1+e^{\frac{t-\tau_{\text{TS}}}{\tau_{trans}}}} \end{array}$$

*P* is the transition probability, *t* is simulation time, τ_*TS*_ is the fitted time when *P* = 50%, and τ_*trans*_ sets the period when *P* is ranging from 0.4 to 0.6 (Pande and Rokhsar, 1999; Gsponer and Caflisch, 2002; Chong et al., 2005). Average TS structure was selected from TSE near τ_*TS*_ of each trajectory.

## Results

To collect enough snapshots for statistically meaningful structural analysis, six trajectories of 10.0 ns were collected for apo- and polymerase-bound DNA/RNA hybrid with DNA damage. Additionally, bound DNA/RNA hybrid with mismatches was also simulated. Average Cα RMSDs with respect to simulation time (shown in Figure S1) indicate that 10 ns' simulation time at 298 K was sufficient for the three systems to equilibrium in the fully solvated environment. Bound DNA/RNA hybrid shows a lower RMSD than the apo-structure, which reflects a stabilizing role of the binding between the two components.

The average structures of bound and apo DNA/RNA hybrid are shown in Figure S1. The 3′-end of the RNA was extruded by the backtracked protein and the 5′-end of the DNA was incurved. The protein lost some α-helices at the N-terminal and rotated about 30°. Similar changes can be found in the complex of RNA/DNA mismatch.

### Induced fit or conformational selection in polymerase and DNA/RNA recognition

**Figure 2:**
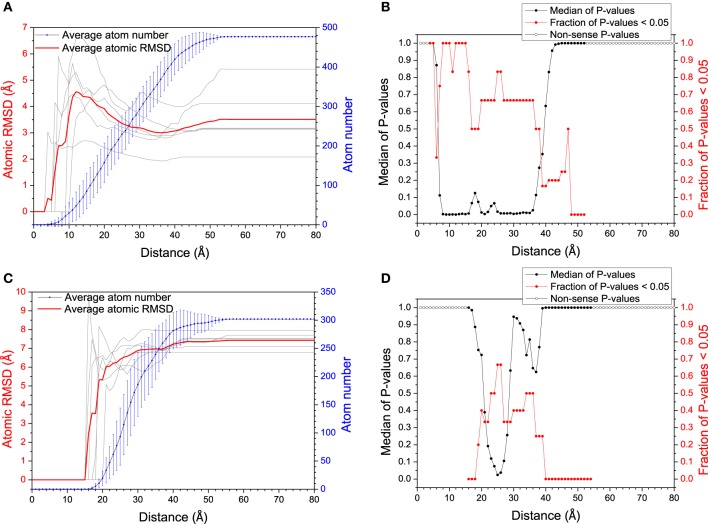
**RMSD (between bound structure and its most similar apo-structure) vs. distance from the mass center of the polymerase (A) and the DNA/RNA hybrid (C)**. Larger RMSD in short distance (larger conformational change around binding site) reflects larger magnitude of mechanism of induced fit. The significance of the conformational change between within every distance from the binding site and within the whole molecule was tested by KS *P*-test, for DNA/RNA hybrid **(B)** and the polymerase **(D)**, respectively.

The mechanism of specific binding is one of the most attractive issues in our study. Recently, residual dipolar coupling experiment suggests that the folding of ubiquitin complex obeys conformational selection rather than induced-fit mechanism (Lange et al., 2008). Nevertheless, both mechanisms have been observed in the same system (James and Tawfik, 2003; Okazaki and Takada, 2008). To evaluate the mechanism of specific recognition between DNA/RNA hybrid and polymerase from MD simulation, Figure 2 shows the average atom RMSD values between the bound structures and its most “like” apo structure vs. the distance from DNA/RNA or polymerase binding site. The RMSD gradually decreases for DNA/RNA hybrid, however, the RMSD of polymerase has the propensity of increases. These observations suggest DNA/RNA adjusts the conformation near the binding site. This conformational changes were then tested by two-sample Kolmogorov-Smirnov (KS) *P*-test (Wlodarski and Zagrovic, 2009) to see the significance of these structural differences. The conformational difference is statistically significant up to ≈30 Å away from the polymerase binding site with the median *P*-values typically < 0.05 and the fraction of typical *P* > 0.5, which suggests that the local conformational changes are significant for DNA/RNA. Therefore, bound DNA/RNA hybrid might obey an induced fit mechanism. However, the conformational difference is not significant for the polymerase which can be indicated with the low fraction with *P* < 0.05 for the whole distance range. Different to DNA/RNA hybrid, induced fit may not so significant on the polymerase.

**Figure 3:**
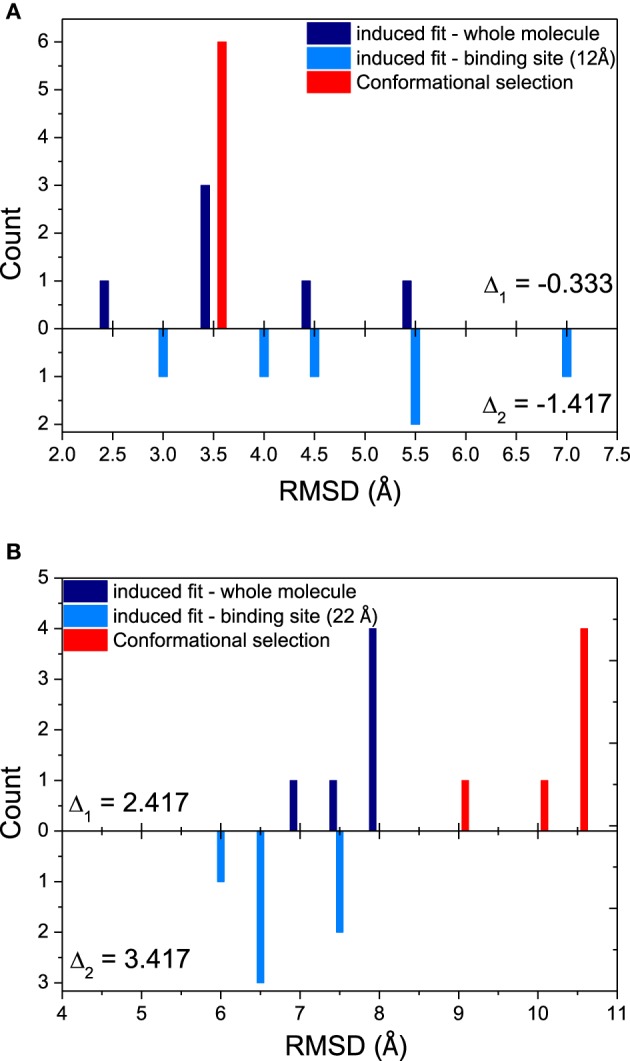
**Relative magnitudes of conformational selection and induced fit were represented in histograms that show counts of quantified RMSDs between: bound structures and most similar apo-structures in whole molecule (global induced fit, dark blue bars); bound structure and most similar apo-structure near binding site (local induced fit, light blue bars); and the most similar apo-structure and other apo-structures (conformational selection, red bars)**. **(A)** DNA/RNA, **(B)** Polymerase. Δ_1_ is calculated from CS−IF_global_, while Δ_2_ means CS−IF_bindingsite_.

Since both mechanisms would exist in the binding process, relative magnitude for these mechanisms is another interesting issue in our study. The histograms comparing the magnitude of conformational selection with induced fit at 12 Å (just the binding site) and 40 Å (the whole molecule) for DNA/RNA hybrid and polymerase, and shown in Figure 3. Parameter Δ represents the difference between conformational selection and induced fit, Δ_1_ for the whole molecule, Δ_2_ for the binding site. For DNA/RNA hybrid, both Δ_1_ and Δ_2_ are negative. This indicates that the magnitude of induced fit is higher than that of conformational selection. That is, the folding of DNA/RNA hybrid might predominantly follow an induced fit mechanism upon the protein binding. Consistent as the observation above, protein might obey different mechanisms from the view of the positive value for both whole molecule and the binding site. Polymerase might follow a conformational selection mechanism when binding onto DNA/RNA hybrid.

### Distance analyses

**Figure 4:**
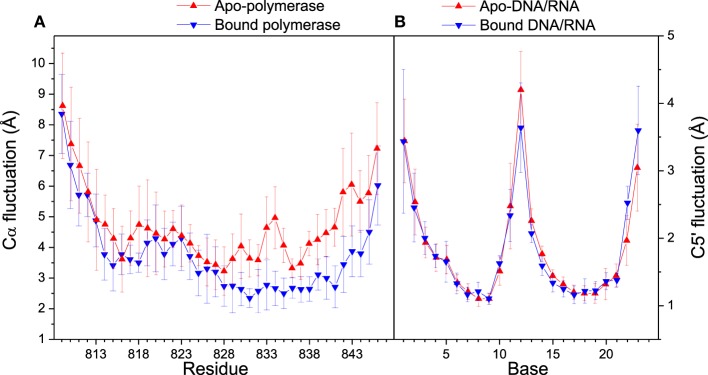
**Cα and C5′ fluctuations in folded state**. **(A)** Comparison of Cα fluctuations between apo- (red) and bound (blue) polymerase; **(B)** Comparison of C5′ fluctuations between apo- (red) and bound (blue) polymerase. Standard deviations among six trajectories are displayed with error bars.

Distance analyses could be used to further confirm the conformation adjustment in the binding process. C5′ and Cα fluctuations for bound and apo states are illustrated in Figure 4. The Cα fluctuation of bound polymerase is significantly lower than that of apo-polymerase, especially in the region of Residue 829–846, which suggests that polymerase becomes more stable upon DNA/RNA binding. The conformations of 3′-end of RNA and 5′-end of DNA for bound DNA/RNA hybrid also have significant change upon the interaction of polymerase.

**Figure 5:**
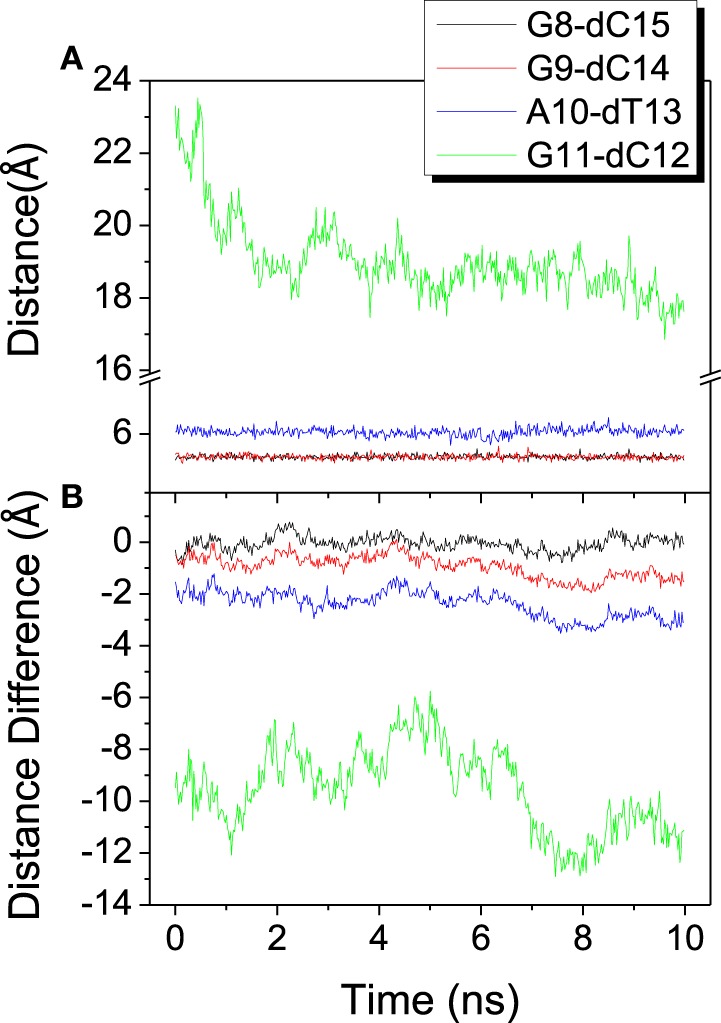
**Distance among the mass center of the polymerase and the last 4 base pairs of the DNA/RNA hybrid**. **(A)** Distance between each base pair. **(B)** Distance difference for each base pair, calculated from distance of RNA to protein minus that of DNA to protein.

To investigate the relative movement in the binding process, distances from last 4 base pairs of bound DNA/RNA hybrid to the mass center of the protein are analyzed and shown in Figure 5. The last base pair (G11-dC12) has the propensity of constriction, however other three base pairs keep stable from each other. From the result of distance difference (Figure 5B), the relative location of protein between DNA and RNA has large fluctuation and keeps dynamics equilibrium. Protein keeps much closer to the G11 of RNA than to dG12 of DNA. This is consistent with the previous work (Gnatt et al., 2001).

**Figure 6:**
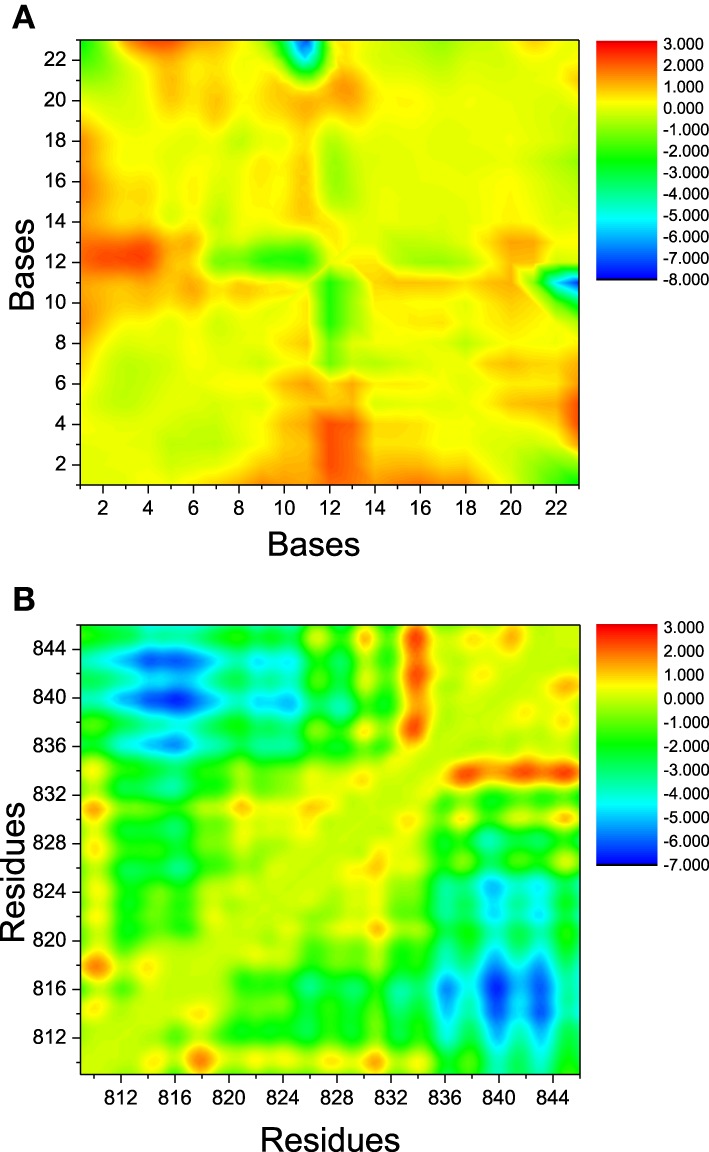
**The landscape of distance difference between bound and apo states**. **(A)** DNA/RNA, **(B)** polymerase. The color in red represents positive distance difference. The color in blue represents negative distance difference.

The landscape of distance difference for base pairs between apo and bound DNA/RNA is shown in Figure 6A. Distance difference landscape can reflect the relative conformational change of the DNA/RNA backbone. The deep red areas indicate that the distance difference for bases dC12-U2, dC12-C3, and dC12-G4 is positive value, which suggests that these base pairs are contracted upon polymerase-binding. The blue areas represent that the distance difference is negative value. It suggests that dC23 of DNA becomes far away from G11 of RNA. The distance landscape for residue pairs between apo and bound polymerase is shown in Figure 6B. The result shows that the most regions of polymerase backbone did not undergo an obvious conformational change except the residue pairs of Glu831/Gln837, Glu831/Val842, Glu831/Leu845, and Phe814, Phe815, His816 with Arg840, Lys843. These regions might play a key role in the formation of DNA/RNA/polymerase complex.

### Driving forces for the conformation adjustment

**Figure 7:**
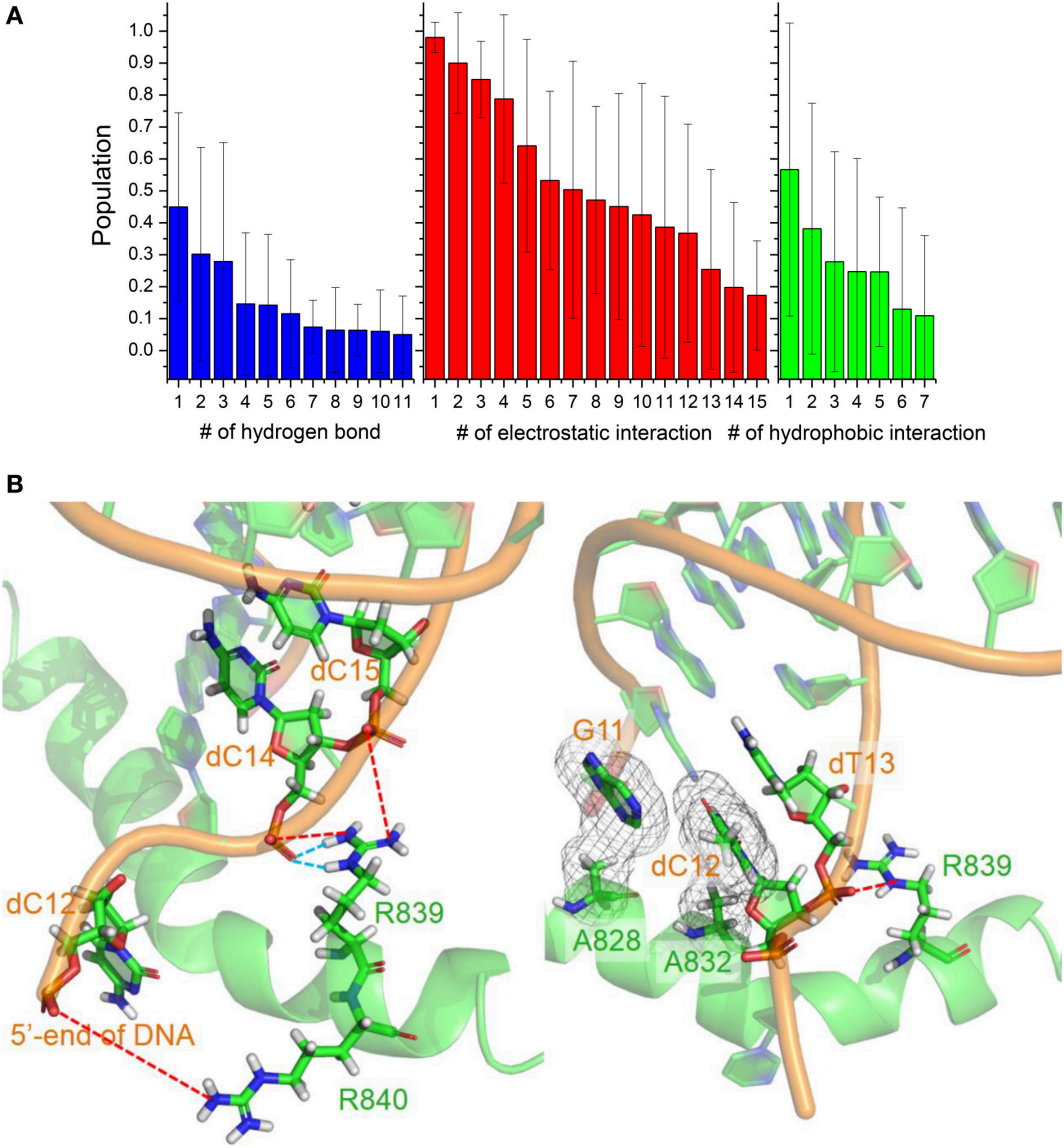
**Interactions between DNA/RNA hybrid and polymerase**. **(A)** Histograms of number of three kinds of interactions. **(B)** Cartoon representations of several important interactions. Blue dashed lines represent the hydrogen bonds, which indicate two stable hydrogen bonds between Arg839 and dC14; Red dashed lines represent the strong electrostatic interactions between positive and negative charged residues; And meshes involve the two stable hydrophobic cores.

The conformation adjustment in the binding process could be attributed to the electrostatic, hydrophobic and hydrogen-binding interactions between DNA/RNA and polymerase. The electrostatic interactions in the simulation of six trajectories are shown in Figure 7. There are 12 electrostatic interactions between positively charged amino acids and the phosphates of DNA/RNA with populations higher than 30%. The positive charged residues, such as Arg821, Arg839, Arg840, and Lys843, provide electrostatic interacts with the phosphates of the DNA/RNA terminal. More than 80% electrostatic interactions are located between polymerase and DNA. The hydrophobic contacts in the simulation of six trajectories are also shown in Figure 7. Two stable hydrophobic interactions can be found: Ala832-dT13 and Ala828-G11, with populations higher than 30%. Figure 7 also shows two hydrogen bonds with populations higher than 30%. These hydrogen bonds are between residues Arg839 and Watson-Crick edge of dC14. Comparing among these three kinds of interactions, electrostatic interactions is in predominance, which suggests that the electrostatic interactions play key roles in the backtracking and proofreading of polymerase.

### Unfolding kinetics

**Figure 8:**
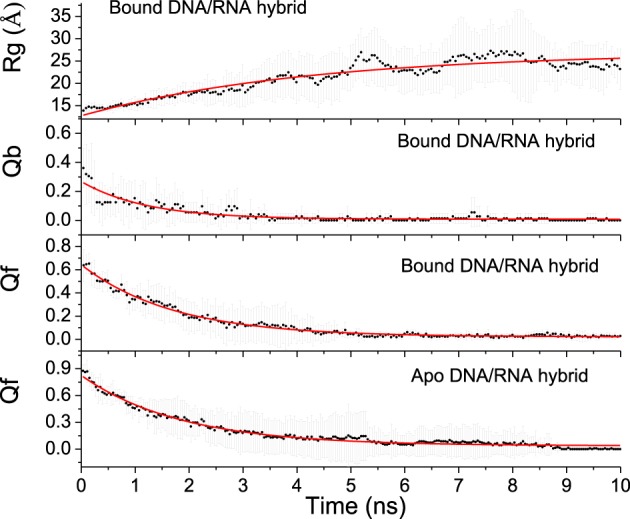
**Fitting of kinetics parameters for bound and apo-DNA/RNA hybrids**.

Unfolding state along high temperature simulations was performed to detect the binding and folding details of DNA/RNA hybrid and the polymerase. The fraction of tertiary native contacts (Qf) and binding native contacts (Qb) is used to monitor unfolding and unbinding kinetics, respectively. The stable native contacts were calculated for the folded state, including inter-chain and intra-chain. Qf is defined as the fraction of intrachain native contacts which are still stable at unfolding state; Similarly, Qb is the fraction of interchain native contacts at 498 K. Time evolutions of Qb and Qf for bound DNA/RNA are shown in Figure 8, which suggests that the tertiary unfolding and unbinding kinetics can be represented well by single exponential functions, indicating first order kinetics in the NVT ensemble at high temperature. The fitted kinetics data are listed in Table 3. The kinetics analysis shows that the unbinding half-time is 1.21 ± 0.08 ns and the tertiary unfolding half-time is 1.71 ± 0.04 ns. This indicates that the unbinding is slightly faster than the tertiary unfolding for bound DNA/RNA. The time evolution of Qf for apo-DNA/RNA is also shown in Figure 8. It is found that the tertiary unfolding of apo-DNA/RNA also obeys first order kinetics, with a half-time of 1.85 ± 0.04 ns, which is a little slower than the bound DNA/RNA. Time evolution of the radius of gyration (Rg) for bound DNA/RNA is also presented in Figure 8. The extension of bound DNA/RNA obeys first order kinetics, and the half-time is 4.24 ± 0.40 ns. This indicates that the extension of bound DNA/RNA is slower than the tertiary unfolding and unbinding. There were 14 native contacts between the DNA and the protein at folded state. These native contacts sharply decreased during unfolding (5 in transition state and only 1 in unfolded state). There were 13 native contacts between the RNA and the enzyme at folded state, but only 2 in transition state. At unfolded state, RNA separated from the enzyme.

**Table 3:** **Unfolding kinetic constants for bound and apo DNA/RNA hybrid**.

**Structure**	**Property**	**τ (ns)**	***A***	***B***	***R*^2^**
Bound DNA/RNA hybrid	Rg	4.24±0.40	−14.22±0.42	26.96±0.50	0.8852
	Qb	1.21±0.08	0.26±0.01	0.01±0.00	0.8490
	Qf	1.71±0.04	0.62±0.01	0.02±0.00	0.9835
Apo-DNA/RNA hybrid	Qf	1.85±0.04	0.79±0.01	0.04±0.00	0.9797

### Unfolding landscape

**Figure 9:**
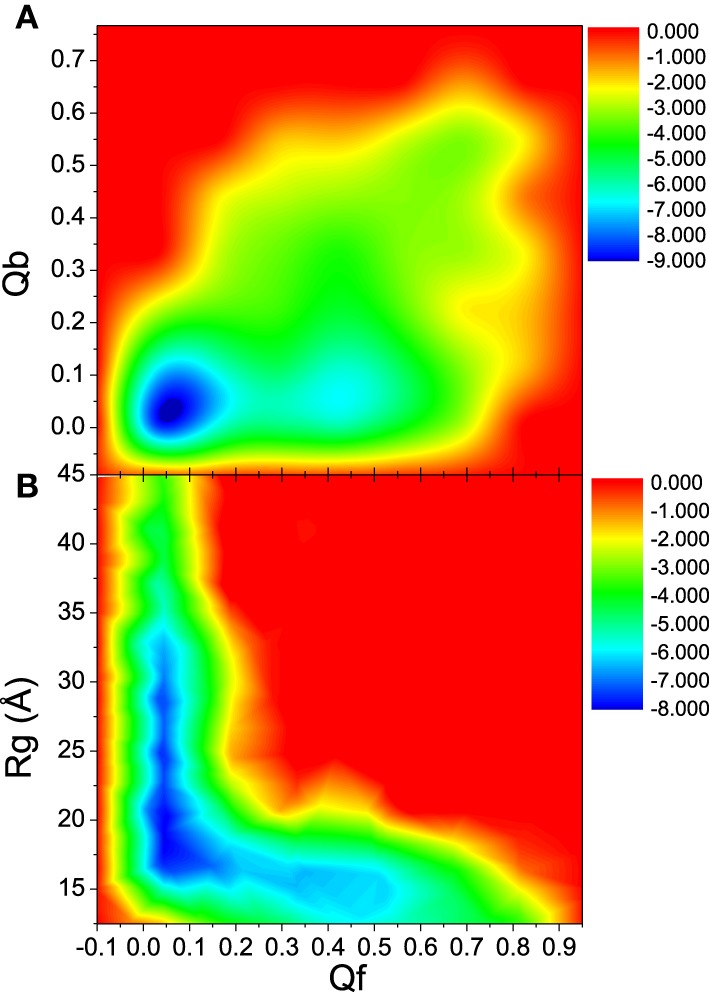
**Unfolding landscape with respect to Qb, Rg, and Qf of bound DNA/RNA hybrid**. **(A)** Landscape of Qb and Qf. **(B)** Landscape of Rg and Qf.

To further understand the interdependence between the DNA/RNA folding and polymerase binding, the unfolding landscape of bound DNA/RNA was analyzed with the variables Qf and Qb, and shown in Figure 9. The unfolding landscape shows that the unbinding proceeds first while the tertiary contacts are held stable, and then followed by the tertiary unfolding. This is consistent with the unfolding kinetics analysis. The formation of the tertiary contacts might be important to the binding of polymerase. The coupling between the tertiary unfolding and the extension of DNA/RNA hybrid is also illustrated in Figure 9. It is found that Qf decreases first while the radius of gyration does not change, and then followed by the extension of DNA/RNA, which suggests that the extension of DNA/RNA is followed by the tertiary unfolding. This is also consistent with the unfolding kinetics analysis.

### Transition state

**Figure 10:**
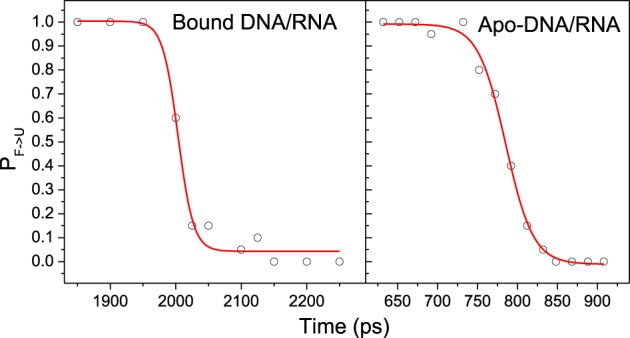
**A representative transition probability ***P*** calculated at 498 K for the F ⇔ U transition for snapshot in the transition region for one of the trajectories of bound and apo-DNA/RNA hybrid, respectively**. The red line is the fit to *P* = 1/{1 + exp [(τ-τ_*TS*_)/ τ_*trans*_]}.

Kinetics analysis shows that the unfolding of bound and apo-DNA/RNA obeys first order kinetics, which suggests that bound and apo-DNA/RNA unfold via a two state process. Therefore, there is a transition state ensemble (TSE) corresponding to the free energy maximum. TSE structures can either fold or unfold, and the transition probability (P) will be 50%. In order to determine TSE, we have scanned MD snapshots for TSE structures in all six unfolding trajectories for each of bound DNA/RNA and apo-DNA/RNA, respectively (Pande and Rokhsar, 1999). The transition probability curves are further fitted by the Boltzmann equation and shown in Figure 10. The analysis yields 50 snapshots for the bound DNA/RNA and 61 snapshots for the apo-DNA/RNA TSE, respectively.

The average structures of TSE for bound and apo states are shown in Figure S2. There are 22 native contacts within bases for bound DNA/RNA TSE and 18 native contacts for apo-DNA/RNA TSE. Apparently, it can be concluded that the TSE of bound DNA/RNA is more native-like than that of apo-DNA/RNA. The stability of the complex is the foundation for backtracking.

### Φ-value prediction

**Figure 11:**
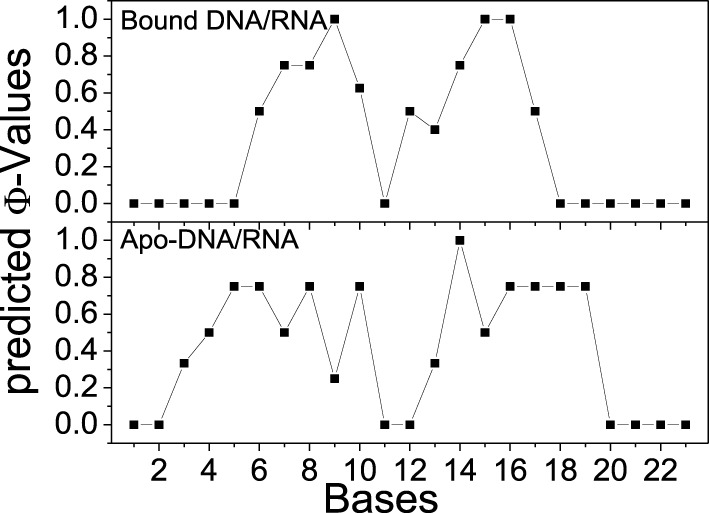
**Predicted Φ-values of bases for bound and apo-DNA/RNA hybrid, respectively**.

Φ-values have been widely used to determine key residues in the protein folding by theoretical and experimental investigations (Fersht, 2000; Fersht and Daggett, 2002; Fernández-Escamilla et al., 2004; Fersht and Sato, 2004). In this study, all TSE structures were used to predict Φ-values of bases for bound and apo-DNA/RNA. The predicted Φ-values of bases for bound and apo-DNA/RNA are shown in Figure 11. Φ-Values of the bases at 3′-end of the RNA for bound DNA/RNA (C7, G9, dC12, dT13, dC15, and dT16), are larger than those for apo-DNA/RNA, which indicates these bases play a key role in the folding of bound DNA/RNA. This is consistent with the interactions analyses that these critical bases of G9, dC12, dT13, and dC15 form electrostatic interactions, native contacts and hydrogen bond network with polymerase. These predicted Φ-values could be further confirmed by experiments.

## Discussion

Three states of RNA polymerase are only different at the translocation of bridge-helix of the polymerase. Most interactions and specific recognitions are between the RNA's 3′-end of DNA/RNA hybrid and the bridge-helix of the polymerase. In molecular modeling and MD simulations, it is quite normal to simplify the system that is object of study to make it more computationally affordable. For the same reason, we selected the core piece of bridge helix, Thr809 to Glu846, which is the most important polypeptide in backtracked polymerase.

### Comparison with experiments

Many experiments released the structural basis of transcription for RNA polymerase II, template DNA, and nascent RNA (Cramer et al., 2000, 2001; Gnatt et al., 2001; Shaevitz et al., 2003; Hahn, 2004; Kettenberger et al., 2004; Wang et al., 2009). The main observation is focused on high electron density at the 3′-end of the RNA, indicating the extrusion and instability of the 3′-terminal of RNA. Gnatt et al. (2001) reported that the specific recognition for RNA polymerase II backtracking may prefer RNA to DNA. In our simulation, RNA polymerase approaches closer to the 3′-end of the RNA than to the 5′-end of the DNA, which is in agreement with the experimental observations.

The structural analyses have shown that Thr831 and Ala832 of polymerase are the critical residues in stabilizing the complex. Our room temperature simulations detected a stable hydrophobic interaction for Ala832/dT13 and two relatively weak hydrophobic interactions (Ala832/dC12 and Ala832/A10) with a population around 25%. For Thr831, there are two stable native contacts (Thr831/A10 and Thr831/G11) with the population about 55%. This is consistent with the observation of X-ray experiment.

### Comparison between DNA damage and DNA/RNA mismatch

Backtracked state could also be caused by the nascent mismatched RNA. In order to compare DNA damage and DNA/RNA mismatch, six trajectories of 10.0 ns each were also simulated for the DNA/RNA mismatch. In the distance analyses (Figure S3), the protein was found to retreat and approach two mismatched base pairs of RNA. Polymerase also moves closer to the 3′-end of RNA than the 5′-end of DNA. This is consistent with the distance analysis for DNA damage.

Interactions between the protein and the mismatched DNA/RNA hybrid were also analyzed and shown in Figure S4. There are 12 stable electrostatic, 4 stable hydrophobic, and 1 hydrogen bond interactions between DNA/RNA and polymerase, with populations higher than 30%. Similar to DNA damage, electrostatic interactions form the main interactions stabilizing the binding between the polymerase and mismatched DNA/RNA hybrid. Also, these non-specific interactions play a key role in recognition and backtracking of the enzyme. Average structure of mismatched DNA/RNA hybrid was also analyzed and shown in Figure S1. This figure suggests that the 5′-end of the DNA was incurved and the 3′-end of the RNA was extruded. This is also in agreement with the structural analysis.

### Comparison with other simulations

**Figure 12:**
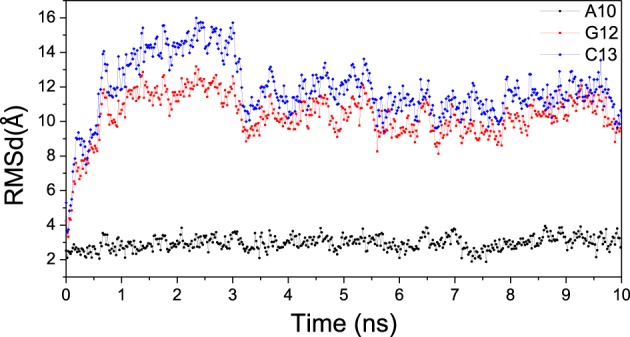
**RMSD for A10, G12, and C13 of the RNA in bound mismatch DNA/RNA**.

The RMSD for A10, G12, and C13 of bound DNA/RNA mismatch was analyzed respectively, and shown in Figure 12. The C5′ RMSD of A10 is the lowest with small fluctuation. For mismatched G12 and C13, the C5′ RMSD is significant high with large fluctuation. This result is qualitatively consistent with the previous simulation (Wang et al., 2009). Furthermore, low stability of mismatched DNA/RNA hybrid leads to easier excision of the nascent and mismatched ribonucleotides for polymerase.

In our simulation, the RMSF of bound polymerase is significant smaller than that of apo polymerase. This is also consistent with the previous work that RMSFs of free polymerase are ranging from 0.6 to 2.3 Å and bound polymerase from 0.5 to 1.3 Å (Suenaga et al., 2006).

### Further discussions on binding and folding pathways

**Figure 13:**
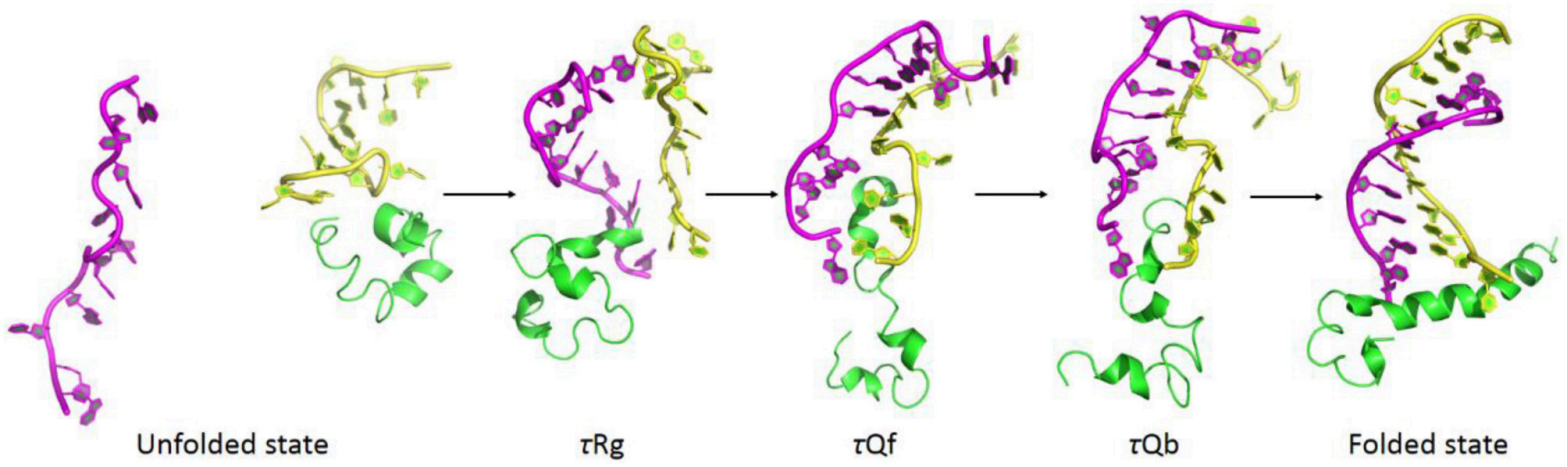
**Average structures along binding and folding pathways of DNA/RNA hybrid, green regions for polymerase, yellow regions for the DNA, and magenta regions for the RNA**. The binding and folding pathways are along (1) > 15 ns (unfolded state), (2) 4.24 ns (τRg), (3) 1.71 ns (τQf), (4) 1.21 ns (τQb), and (5) folded state.

By reversing the unfolding pathway, the binding and folding process could be constructed as (1) unfolded state, (2) tertiary contraction (τRg, 4.24 ns), (3) tertiary folding (τQf, 1.71 ns), (4) tertiary binding (τQb, 1.21 ns), and (5) folded state. Average structures along binding and folding pathway are shown in Figure 13. In the unfolded state, RNA was far away from the complex of DNA and the polymerase. After its binding onto polymerase (τRg), G9 and G11 kept tightly interacting with the polymerase. G9 was considered as a key base from the results of interaction analyses and Φ-value prediction because of the electrostatic interaction between it and the polymerase. This observation also indicate that the binding process is initiated by the non-specific electrostatic interaction to get the RNA and the protein approach to each other, then is stabilized with the specific interactions (hydrogen bonding and hydrophobic interactions). Upon binding, DNA/RNA hybrid undergoes a significant conformational adjustment with these interaction. Then 3′-end of the RNA was extruded by the polymerase, which is the key step in the nascent RNA's cleavage and excision.

## Conclusions

Both 298 K and 498 K molecular dynamics simulations are performed for bound and apo DNA/RNA hybrid. At folded state, conformation of bound DNA/RNA hybrid shows significant adjustment and becomes more unstable upon the polymerase binding, especially the 3′-end of the RNA. Kinetics analysis of MD simulations at 498 K shows that bound and apo DNA/RNA hybrid unfold via a two-state process. Bound DNA/RNA hybrid folds in the order of DNA/RNA contracting, the tertiary folding, and the protein binding. C7, G9, dC12, dC15, and dT16 are key bases to bound DNA/RNA hybrid folding. Inter-chain interaction analyses and binding pathway construction show that the recognition is in the order of non-specific closing, specific binding and conformation adjustment. dC14, G9, A10, and G11 are key bases to the protein recognition. The average RMSD values between the bound structures and the most “like” apo-structure and Kolmogorov-Smirnov (KS) *P*-test analyses indicate that the DNA/RNA hybrid might follow an induced fit mechanism and the polymerase with the conformational selection in their binding process. Furthermore, this method could be used to relative studies of specific recognition between nucleic acid and protein.

### Conflict of interest statement

The authors declare that the research was conducted in the absence of any commercial or financial relationships that could be construed as a potential conflict of interest.
