# Supplementary material for: Conformational selection and induced fit for RNA polymerase and RNA/DNA hybrid backtracked recognition
Source: Front Mol Biosci. 2015 Nov 5;2:61. doi: 10.3389/fmolb.2015.00061 (PMC4633505; doi:10.3389/fmolb.2015.00061)
Supplement: Supplementary file 1 [file DataSheet1.PDF]

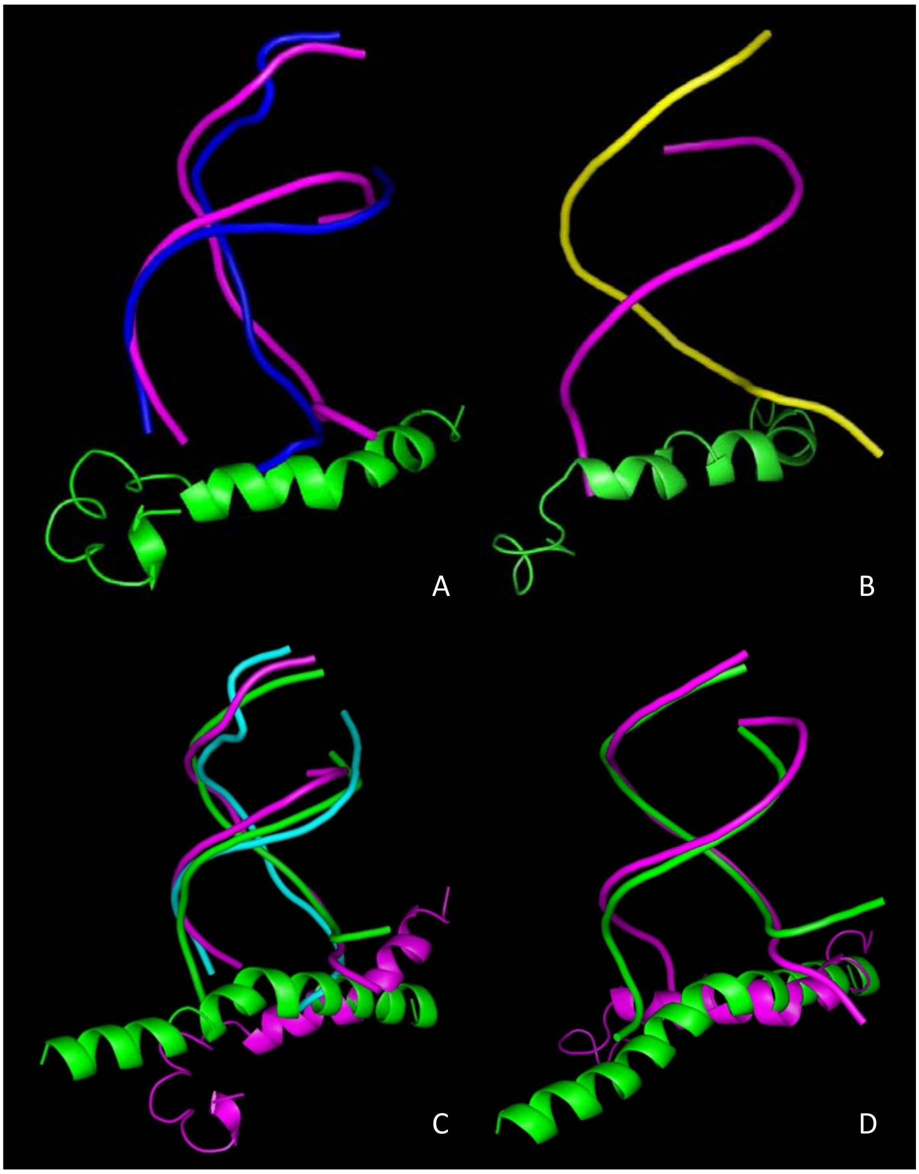

Figure S1. The average structures of bound RNA/DNA damage, Apo-DNA/RNA damage and bound DNA/RNA mismatch. (A) Average structure of bound and apo DNA/RNA damage. green region and magenta region for the polypeptide and the nucleotides in bound DNA/RNA; blue region for the nucleotides in apo DNA/RNA. (B) Average structure of bound DNA/RNA mismatch. green region, magenta region and yellow region for polypeptide, RNA and DNA, respectively. (C) Comparison among the initial structure of bound DNA/RNA (green), the average structure of bound DNA/RNA (magenta) and the average structure of apo DNA/RNA (cyan). (D) Comparison between the initial structure (green) and the average structure (magenta) of bound DNA/RNA mismatch.

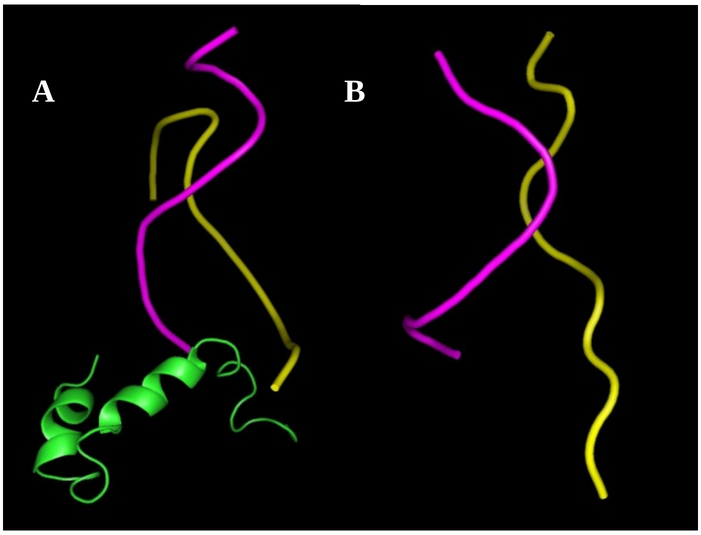

Figure S2. Average TSE structure for bound (A) and apo-DNA/RNA (B). Green region represents the enzyme, yellow region for DNA and magenta region for RNA.

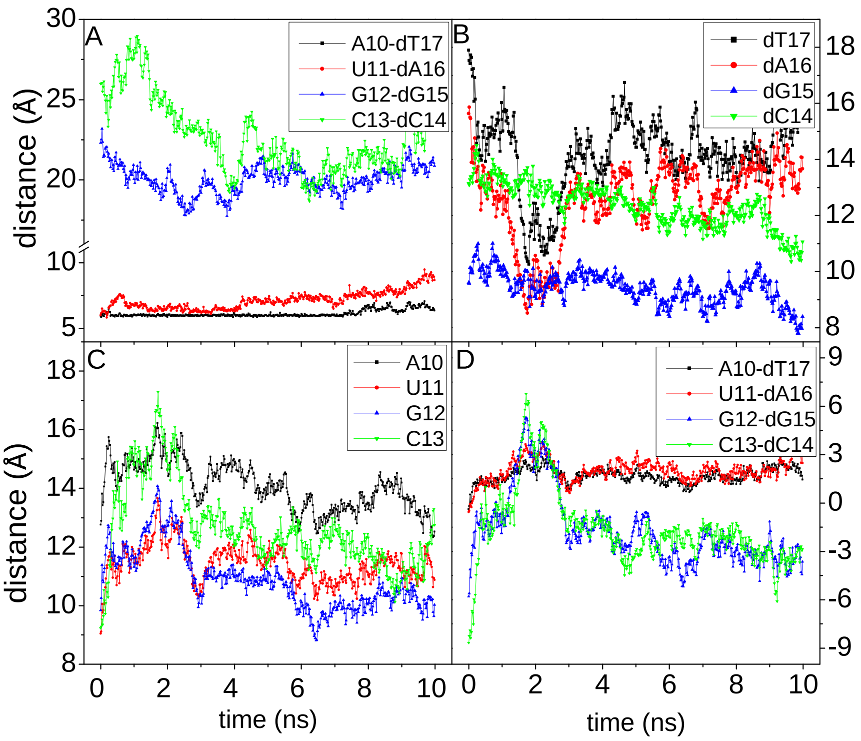

Figure S3. Distance changes among DNA, RNA and protein for bound DNA/RNA mismatch. (A) Distance between base pair for four pairs of bases. (B) Distance between DNA base and the protein for four bases. (C) Distance between RNA base and the protein for four bases. (D) Distances differences between RNA-protein and DNA-protein.

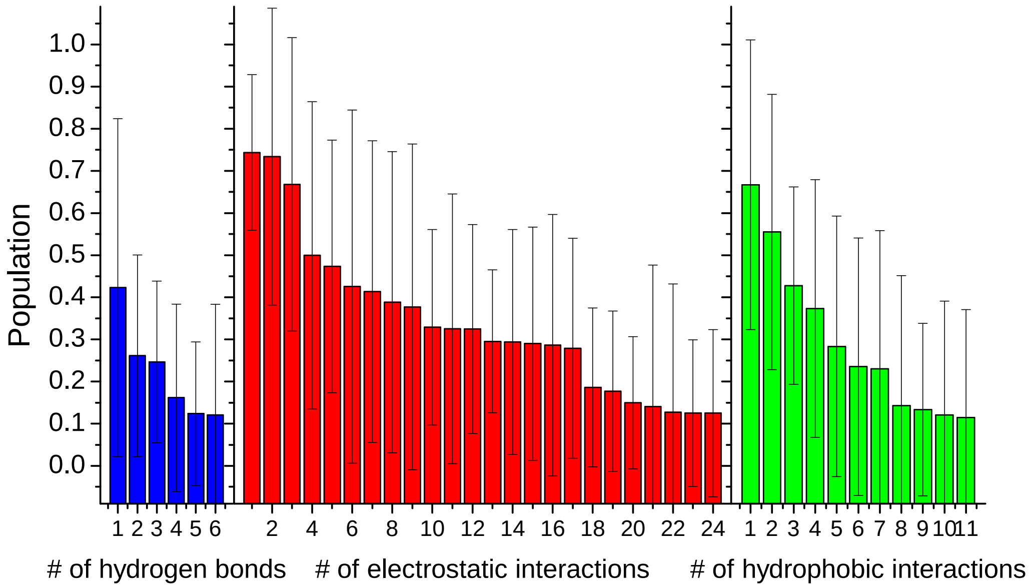

Figure S4. Interactions between protein and DNA-RNA hybrid for bound DNA/RNA mismatch.
